# Supplementary material for: Complexome profiling of the Chlamydomonas psb28 mutant reveals TEF5 as an early PSII assembly factor
Source: Plant Cell. 2025 Mar 17;37(6):koaf055. doi: 10.1093/plcell/koaf055 (PMC12164590; doi:10.1093/plcell/koaf055)
Supplement: koaf055_Supplementary_Data [file koaf055_supplementary_data.zip › psb28_interactive.zip!/plots/intensity/qualityPlots/peptides/CPLD31_Cre15.g636050.t1.2.html]

### Cre15.g636050.t1.2

Trivial name: CPLD31  
SeqCoverage [median]: 15.40  
Mass: 26.59 kDa  
  
 

| Sequence | MissedCleavage | Mass [Da] | wtIntensity | muIntensity | Score |
| --- | --- | --- | --- | --- | --- |
| ATSGPFGK | 0 | 763.39 | 498.81 | 855.26 | 81.78 |
| IITEVSNLEK | 0 | 1144.63 | 0.00 | 1871.69 | 101.64 |
| VLAQNYPETPGLAIR | 0 | 1640.89 | 2800.03 | 6949.85 | 205.07 |
|  |  |  | 3298.8 | 9676.8 |  |
